# Association between C reactive protein and microvascular and macrovascular dysfunction in sub-Saharan Africans with and without diabetes: the RODAM study

**DOI:** 10.1136/bmjdrc-2020-001235

**Published:** 2020-07-14

**Authors:** Charles Frederick Hayfron-Benjamin, Anke H Maitland-van der Zee, Bert-Jan van den Born, Albert G B Amoah, Karlijn A C Meeks, Kerstin Klipstein-Grobusch, Matthias B Schulze, Joachim Spranger, Ina Danquah, Liam Smeeth, Erik J A J Beune, Frank Mockenhaupt, Charles O Agyemang

**Affiliations:** 1 Vascular Medicine, Respiratory Medicine and Public Health, Amsterdam University Medical Centres, Amsterdam, The Netherlands; 2 Physiology, University of Ghana Medical School, Accra, Ghana; 3 Anaesthesia, Korle-Bu Teaching Hospital, Accra, Ghana; 4 Respiratory Medicine, Amsterdam University Medical Centres, Amsterdam, The Netherlands; 5 Internal Medicine, Amsterdam University Medical Centres - Locatie AMC, Amsterdam, The Netherlands; 6 Medicine and Therapeutics, University of Ghana Medical School, Accra, Ghana; 7 Public Health, Amsterdam University Medical Centres, Amsterdam, The Netherlands; 8 Center for Research on Genomics and Global Health, National Human Genome Research Institute, Bethesda, Maryland, USA; 9 Julius Global Health, Julius Centrum voor Gezondheidswetenschappen en Eerstelijns Geneeskunde, Utrecht, The Netherlands; 10 Epidemiology and Biostatistics, School of Public Health, Faculty of Health Sciences, University of the Witwatersrand, Johannesburg, South Africa; 11 Molecular Epidemiology, German Institute of Human Nutrition Potsdam-Rehbruecke, Nuthetal, Germany; 12 Endocrinology and Metabolism, Charité Universitätsmedizin Berlin, Berlin, Germany; 13 Center for Cardiovascular Research (CCR), Charite-Universitaetsmedizin Berlin, Berlin, Germany; 14 Heidelberg Institute of Global Health, University of Heidelberg, Heidelberg, Germany; 15 Non-communicable Disease Epidemiology, London School of Hygiene and Tropical Medicine, London, London, UK; 16 Institute of Tropical Medicine and International Health, Charité Universitätsmedizin Berlin, Berlin, Germany

**Keywords:** C-reactive protein, inflammation, microvascular and macrovascular complications, adult diabetes

## Abstract

**Introduction:**

Although inflammation assessed by elevated C reactive protein (CRP) concentration is known to be associated with risk of cardiovascular disease, its association with microvascular and macrovascular dysfunction in diabetes and non-diabetes remains unclear. We examined the association between CRP and diabetes and associated microvascular and macrovascular dysfunction in sub-Saharan Africans with and without diabetes.

**Research design and methods:**

Cross-sectional analyses of baseline data from the multicenter RODAM study (Research on Obesity and Diabetes among African Migrants) including 5248 Ghanaians (583 with diabetes, 4665 without diabetes) aged 25–70 years were done. Logistic regression analyses were used to examine the associations between CRP Z-scores and diabetes and microvascular (nephropathy) and macrovascular (peripheral artery disease (PAD)) dysfunction, with adjustments for age, sex, site of residence, smoking, body mass index, systolic blood pressure, and low-density lipoprotein cholesterol.

**Results:**

In the fully adjusted models, higher CRP concentration was significantly associated with diabetes (adjusted OR 1.13; 95% CI 1.05 to 1.21, p=0.002). In participants with diabetes, higher CRP concentration was associated with PAD (1.19; 1.03 to 1.41, p=0.046) but not nephropathy (1.13; 0.97 to 1.31, p=0.120). Among participants without diabetes, higher CRP concentration was associated with higher odds of PAD (1.10; 1.01 to 1.21, p=0.029) and nephropathy (1.12; 1.04 to 1.22, p=0.004).

**Conclusions:**

In this study, higher CRP concentration was associated with higher odds of diabetes in sub-Saharan Africans. Also, higher CRP concentration was associated with higher odds of nephropathy and PAD in non-diabetes and higher odds of PAD in diabetes. CRP may be an important marker for assessment of risk of diabetes and risk for PAD and nephropathy in sub-Saharan Africans with and without diabetes.

**Significance of this study**

**What is already known about this subject?**

- In European and Asian populations, inflammation measured by elevated C reactive protein (CRP) is known to be associated with diabetes.
- Inflammation is a known factor in the development of atherosclerosis and subsequent atherosclerotic vascular events.

**What are the new findings?**

- In sub-Saharan Africans, higher CRP concentration is associated with higher odds of diabetes, even after adjustments for the conventional cardiovascular risk factors.
- Higher CRP concentration is significantly associated with higher odds of peripheral artery disease (PAD) and nephropathy in non-diabetes and higher odds of PAD in diabetes.
- The conventional cardiometabolic risk factors did not explain the associations between CRP and PAD and nephropathy in diabetes and non-diabetes.

**How might these results change the focus of research or clinical practice?**

- Our findings suggest that CRP could be explored as a potential marker to identify sub-Saharan Africans at risk of diabetes, PAD and nephropathy.

## Introduction

Globally, microvascular and macrovascular diseases are important public health problems.^1–4^ In many regions of the world including sub-Saharan Africa, the rates of microvascular and macrovascular diseases are rising, contributing to the increasing rates of disability and death from cardiovascular disease (CVD).^5 6^ Specifically, macrovascular diseases including peripheral artery disease (PAD), coronary artery disease, and cerebrovascular disease may complicate critical limb ischemia, myocardial infarction, and stroke, respectively.^7^ Also, microvascular diseases such as retinopathy, nephropathy, and neuropathy may result in blindness, end-stage kidney disease, and lower limb amputation, respectively.^8^

C reactive protein (CRP), the most extensively studied biomarker of inflammation, is known to be significantly associated with CVD including diabetes in European and Asian populations; however, data on its role in CVD in sub-Saharan African populations are limited.^9 10^ Considering the substantial ethnic differences in the association between inflammation and diabetes, it may be valuable to investigate this association in sub-Saharan African populations, in the quest to integrate CRP to global risk scores for diabetes.^11^

Existing data on the association between inflammation and vascular dysfunction have focused on individuals with diabetes.^12–14^ In diabetes, endothelial injury from inflammation mediated by chronic hyperglycemia is known to play a key role in the development of vascular complications.^4 15^ However, the effect of glycemic control on macrovascular complication risk or progression in diabetes remains uncertain.^13 16^ Also, existing data suggest important associations between inflammation and CVD in non-diabetes.^17^ Given the above, it is plausible that inflammation triggered by causes other than hyperglycemia may be important in the pathogenesis of microvascular and macrovascular dysfunction; however, the biological basis for this association has not been clarified.^17^ We, therefore, assessed the associations between CRP and diabetes in Ghanaians. In addition, we assessed associations between CRP and microvascular (nephropathy) and macrovascular dysfunction (PAD) in Ghanaians with and without diabetes.

## Materials and methods

### Study design

**Figure 1:**
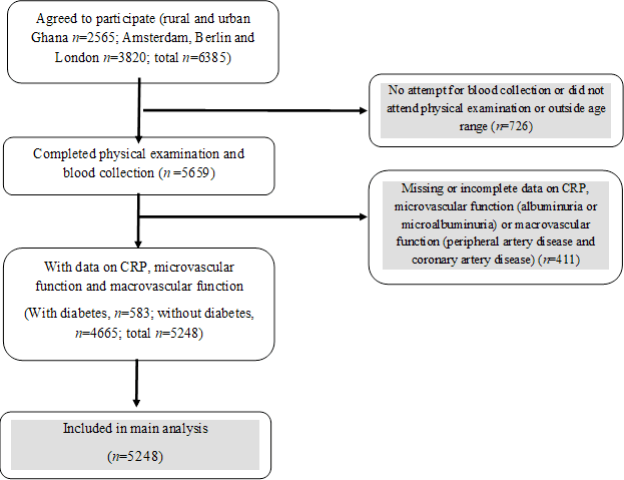
Flow chart of study design and inclusion in analyses. CRP, C reactive protein.

The rationale, conceptual framework, design and methodology of the RODAM study (Research on Obesity and Diabetes among African Migrants) have been described in detail elsewhere.^18^ In brief, the study was conducted from 2012 to 2015 and comprised Ghanaians aged 25–70 years living in rural and urban Ghana as well as in three European cities, namely Amsterdam, Berlin and London. Data collection for the study was standardized across all sites. Informed written consent was also obtained from each participant prior to enrollment in the study. For the current analyses, only participants with complete data on CRP, microvascular disease (nephropathy) and macrovascular disease (PAD) were included. This comprised data from 583 participants with diabetes and 4665 participants without diabetes aged 25–70 years (figure 1).

### Assessments

#### Questionnaires

A structured questionnaire^18^ was used to record the demographic, socioeconomic, and health-related behaviors of the study participants. Smoking was assessed as a positive reply to the question ‘Do you smoke at all?’ Alcohol intake in grams per day was estimated using standard portion sizes combined with frequencies of intake based on a standardized Food Propensity Questionnaire.^19^ Physical activity was derived for each participant using the International Physical Activity Questionnaire, and participants were classified into three categories of total physical activity, namely low, moderate and high level.^20^

#### Physical examination

According to standard operation procedures, weight was measured in light clothing and without shoes with SECA 877 scales to the nearest 0.1 kg. Height was measured without shoes with SECA 217 stadiometer to the nearest 0.1 cm. Body mass index (BMI) was calculated as weight (kg) divided by height squared (m^2^). Blood pressure (BP) was measured three times using the Microlife WatchBP home device, with appropriately sized cuffs after at least 5 min rest while seated. The mean of the last two BP measurements was used for the analyses. Hypertension was defined as systolic BP ≥140 mm Hg and/or diastolic BP ≥90 mm Hg, and/or being on antihypertensive medication treatment. All the anthropometrics were measured twice by the same assessor and the average of the two measurements was used for analyses.

Ankle-brachial pressure index (ABI) measurements were performed in supine position using a validated oscillometric device (WatchBP Office ABI, Microlife, Widnau) with appropriately sized cuffs after at least 10 min of supine rest.^21^ Systolic BP was measured twice in the right and left brachial artery and twice in the right and left posterior tibial arteries. ABI was calculated by taking the highest arm systolic BP as the denominator and the lowest ankle BP as the numerator.^22^ The lowest of the left and right ABI measurements was used for analyses. ABI obtained by the oscillometric method using the Microlife WatchBP Office ABI has been shown to correlate well with ABI acquired by Doppler ultrasound with 95% agreement between the two methods in diagnosing PAD.^23^

#### Biochemical analyses

According to standard operation procedures, fasting venous blood samples were processed and aliquoted into Sarstedt tubes after collection, registered and then temporarily stored at the research site at −20°C. Aliquoted blood samples and first early morning urine sample were transported to the local research centers, where they were checked, registered and stored at −80°C before being shipped to the central laboratory at Charité-University Medicine Berlin (Berlin, Germany) for determination of biochemical variables. Shipping of the samples from European sites was carried out using Styrofoam boxes filled with dry ice and from Ghana in dry shippers filled with liquid nitrogen. Extensive quality checks were done during the biochemical analysis, including blinded serial measurements. Fasting glucose, total cholesterol, low-density lipoprotein (LDL) cholesterol, high-density lipoprotein cholesterol, and triglycerides levels were determined using the ABX Pentra 400 Chemistry Analyzer (HORIBA ABX, Montpellier, France). Fasting plasma glucose concentration was measured using an enzymatic method (hexokinase). The concentration of total cholesterol was assessed using colorimetric test kits. Glycosylated hemoglobin (HbA_1c_) was measured by high-performance liquid chromatography (Tosoh G8 HPLC Analyzer). The concentration of urinary albumin (in µmol/L) was measured by an immunochemical turbidimetric method (Roche Diagnostics) and urinary creatinine concentration (in μmol/L) was measured by a kinetic spectrophotometric method (Roche Diagnostics). Urinary albumin to creatinine ratio (ACR; expressed in mg/mmol) was calculated by taking the ratio between urinary albumin and urinary creatinine. Serum creatinine concentration was determined by a kinetic colorimetric spectrophotometric isotope dilution mass spectrometry calibration method (Roche Diagnostics). The estimated glomerular filtration rate (eGFR) was calculated using the 2009 CKD-EPI (Chronic Kidney Disease Epidemiology Collaboration) creatinine equation. High-sensitive CRP (hs-CRP) levels were measured in heparin plasma by a particle enhanced immunoturbidimetric assay. Human CRP agglutinates with latex particles were coated with monoclonal anti-CRP antibodies. The aggregates were determined turbidimetrically using Pentra 400 Chemistry Analyzer (HORIBA ABX). Z-scores were computed for each participant based on the participant’s CRP concentration (x), mean CRP concentration (μ) and SD (σ). We computed the Z-scores as: Z-score=(x−μ)/σ.

#### Definition of diabetes and microvascular and microvascular dysfunction

Diabetes was defined according to the WHO diagnostic criteria (self-reported diabetes, documented use of glucose-lowering medication(s), fasting plasma glucose ≥7.0 mmol/L) or HbA_1c_ ≥6.5% or ≥48 mmol/mol.^24^ PAD was defined as ABI ≤0.90.^22^ ABI >1.3 was not considered normal as it could be suggestive of non-compressible vessels.^22^ Albuminuria and eGFR were classified according to the 2012 Kidney Disease: Improving Global Outcomes (KDIGO) guidelines.^25^ eGFR was classified as follows: G1, 90 mL/min/1.73 m^2^ (normal kidney function); G2, 60–89 mL/min/1.73 m^2^ (mildly decreased); G3a, 45–59 mL/min/1.73 m^2^ (mildly to moderately decreased); G3b, 30–44 mL/min/1.73 m^2^ (moderately to severely decreased); G4, 15–29 mL/min/1.73 m^2^ (severely decreased); and G5, <15 mL/min/1.73 m^2^ (kidney failure). Low eGFR was defined as eGFR <60 mL/min/1.73 m^2^ (category ≥G3).^26^ Albuminuria classifications were derived from ACR and were as follows: A1, <3 mg/mmol (normal to mildly increased); A2, 3–30 mg/mmol (moderately increased); and A3, >30 mg/mmol (severely increased). Because of the small number of participants in the severely increased albuminuria category (A3), we combined the moderately increased (A2) and severely increased (A3) categories.^25 26^ Nephropathy was defined as albuminuria and/or eGFR <60 mL/min/1.73 m^2^ based on the KDIGO guidelines.^25 27^

### Statistical analysis

Data were analyzed using the IBM SPSS V.23 for Windows. Data with normal distributions were presented as mean±SD, whereas those not normally distributed presented as median (IQR). Categorical data were presented as frequencies (percentages). No significant interaction between site of residence and CRP was found; therefore, we did not stratify the analyses by site of residence. However, we used site as a covariate in our logistic regression models. Logistic regression analyses were used to examine the associations between CRP concentrations and diabetes, PAD and nephropathy with adjustments for covariates. ORs and their corresponding 95% CIs were estimated. The minimal sufficient adjustment sets for estimating the direct effect of CRP on PAD and nephropathy were determined by a directed acyclic graph (DAG) (online supplementary figure 1; DAG available at dagitty.net/mGETVa3). DAG was chosen because the traditional methods of adjusting for potential confounders may introduce conditional associations and bias rather than minimize it.^28^ Based on the DAG, the minimal sufficient adjustment sets for estimating the total effect of CRP on vascular dysfunction were age, smoking, obesity, hypertension, diabetes and dyslipidemia. Three models were used to examine the data. Model 1 was unadjusted, model 2 was adjusted for age and sex, and in model 3 we additionally adjusted for site of residence, smoking, BMI, systolic BP, diabetes and LDL-cholesterol. In the diabetes and non-diabetes subgroup analyses, we adjusted for age, sex, site of residence, smoking, BMI, systolic BP, and LDL-cholesterol in model 3.

10.1136/bmjdrc-2020-001235.supp1 Supplementary data

## Results

### Baseline characteristics

Table 1 shows the baseline characteristics of the study participants. Compared with participants without diabetes, participants with diabetes were less frequently female, less physically active, much older, consumed more alcohol and had a lower proportion of people who have never smoked. Also, the mean BMI, waist circumference, hip circumference, waist to hip ratio, systolic BP, diastolic BP, total cholesterol and LDL-cholesterol were higher in participants with diabetes than their peers without diabetes. The median CRP concentration and proportion of participants with CRP >3 mg/L were higher in participants with diabetes than those without diabetes. Participants with diabetes had higher rates of PAD and nephropathy than their peers without diabetes.

**Table 1:** Baseline characteristics, cardiometabolic risk profiles and rates of microvascular/macrovascular disease according to diabetes status

	Whole cohort (n=5248)	Non-diabetes (n=4665)	Diabetes (n=583)	P value
Female	3263 (62.2%)	2938 (63.0%)	325 (55.7%)	0.001
Age, years	46.16 (±11.48)	45.25 (±11.72)	52.65 (±9.73)	<0.001
Higher or tertiary education	550 (10.7%)	486 (10.6%)	64 (11.2%)	0.578
Physical activity				<0.001
Low level	1423 (30.6%)	1225 (29.6%)	198 (38.7%)	
Moderate level	917 (19.7%)	812 (19.6%)	105 (20.5%)	
High level	2306 (49.6%)	2099 (50.7%)	207 (40.5%)	
Body mass index, kg/m^2^	26.79 (±5.36)	26.76 (±5.37)	29.18 (±5.84)	<0.001
Waist circumference, cm	90.18 (±12.64)	89.23 (±12.26)	97.80 (±13.08)	<0.001
Hip circumference, cm	100.33 (±11.65)	99.92 (±11.57)	103.65 (±11.76)	<0.001
Waist to hip ratio	0.90 (±0.07)	0.89 (±0.07)	0.94 (±0.07)	<0.001
Alcohol consumption, g/day	0.14 (1.99)	0.14 (1.87)	0.58 (3.14)	0.067
Smoking status				<0.001
Current smokers	151 (2.9%)	135 (2.9%)	16 (2.8%)	
Previous smokers	375 (7.2%)	303 (6.6%)	72 (12.4%)	
Never smoked	4667 (89.9%)	4176 (90.5%)	491 (84.8%)	
Systolic BP, mm Hg	129.38 (±19.55)	129.12 (±19.35)	138.11 (±19.47)	<0.001
Diastolic BP, mm Hg	81.08 (±11.97)	80.86 (±11.94)	84.80 (±11.31)	<0.001
Hypertension	2391 (45.6%)	1952 (41.8%)	439 (75.3%)	<0.001
HbA_1c_, mmol/mol	37.93 (±12.90)	35.61 (±6.06)	59.30 (±22.97)	<0.001
Glucose, mmol/L	5.40 (±1.96)	5.01 (±0.58)	8.19 (±4.25)	<0.001
Total cholesterol, mmol/L	5.02 (±1.14)	4.94 (±1.10)	5.19 (±1.33)	<0.001
Triglycerides, mmol/L	1.03 (±0.56)	0.98 (±0.53)	1.25 (±0.73)	<0.001
LDL-cholesterol, mmol/L	3.22 (±0.99)	3.16 (±0.95)	3.34 (±1.18)	<0.001
HDL-cholesterol, mmol/L	1.33 (±0.36)	1.34 (±0.36)	1.29 (±0.34)	0.003
CRP concentration, mg/L	0.70 (2.30)	0.70 (2.10)	1.50 (4.40)	<0.001
CRP categories				<0.001
CRP ≤3 mg/L	4124 (78.6%)	3734 (80.0%)	390 (66.9%)	
CRP >3 mg/L	1124 (21.4%)	931 (20.0%)	193 (33.1%)	
ACR, mg/mmol	0.57 (0.73)	0.55 (0.67)	0.80 (1.37)	<0.001
ACR ≥3 mg/mmol	471 (9.0%)	373 (8.0%)	98 (16.8%)	<0.001
eGFR, mL/min/1.73 m^2^	95.26 (±20.18)	90.61 (±19.68)	95.84 (±20.17)	<0.001
eGFR <60 mL/min/1.73 m^2^	165 (3.1%)	130 (2.8%)	35 (6.0%)	<0.001
Nephropathy	593 (11.3%)	470 (10.1%)	123 (21.1%)	<0.001
ABI (left side)	1.16 (±0.13)	1.16 (±0.13)	1.18 (±0.13)	0.003
ABI (right side)	1.15 (±0.13)	1.15 (±0.13)	1.17 (±0.13)	0.036
ABI categories				0.022
≤0.90	312 (5.9%)	273 (5.9%)	39 (6.7%)	
0.91–1.30	4711 (89.8%)	4204 (90.1%)	507 (87.0%)	
>1.30	225 (4.3%)	188 (4.0%)	37 (6.3%)	

Values for categorical variables are given as number (percentage), and for continuous variables as mean (±SD) or median (IQR).

ABI, ankle-brachial pressure index; ACR, albumin to creatinine ratio; BP, blood pressure; CRP, C reactive protein; eGFR, estimated glomerular filtration rate; HbA_1c_, glycosylated hemoglobin; HDL, high-density lipoprotein; LDL, low-density lipoprotein.

### Association between CRP and diabetes

**Figure 2:**
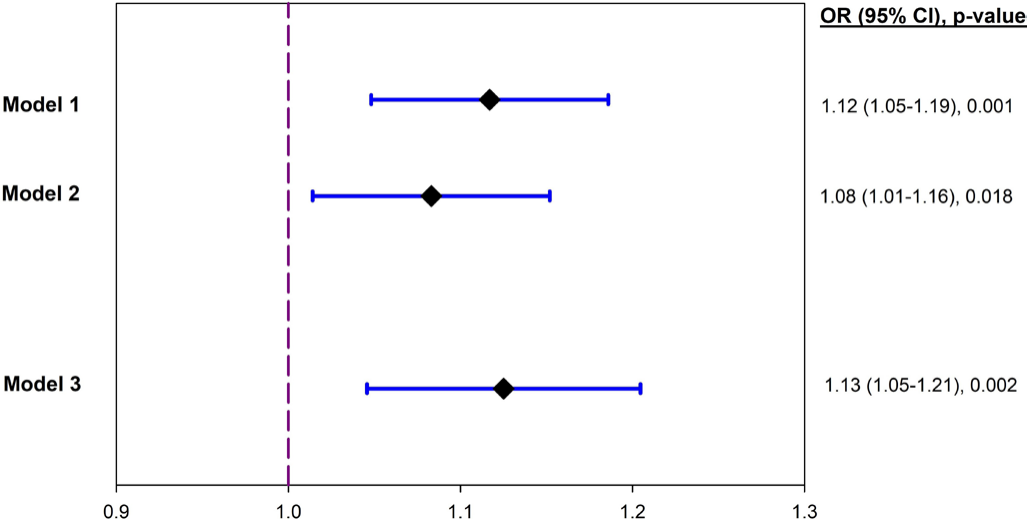
OR of diabetes per SD increase in the CRP concentration. Model 1 was unadjusted; model 2 adjusted for age and sex; model 3 adjusted additionally for site of residence, smoking, BMI, systolic blood pressure, and LDL-cholesterol. SD of mean CRP=8.20 mg/L for the whole group (n=5248); SD of mean CRP=2.07 mg/L for the subgroup of participants with CRP concentrations ≤10 mg/L (n=4962). BMI, body mass index; CRP, C reactive protein; LDL, low-density lipoprotein.

Higher CRP Z-score concentration was associated with higher odds of diabetes in the unadjusted model (figure 2). The association persisted in a fully adjusted model (adjusted OR (AOR) 1.13; 95% CI 1.05 to 1.21, p=0.002). In a sensitivity analysis, we excluded participants with CRP concentrations >10 mg/L; the SD of the mean CRP was 8.20 mg/L for the whole group and 2.07 mg/L for the subgroup with CRP concentrations ≤10 mg/L. In participants with CRP concentrations ≤10 mg/L (n=4962), higher CRP Z-score concentration was significantly associated with higher odds of diabetes in both the unadjusted (OR 1.31; 95% CI 1.22 to 1.42, p<0.001) and fully adjusted (AOR 1.24; 95% CI 1.14 to 1.36, p<0.001) models.

### Association between CRP and microvascular and macrovascular dysfunction

In participants without diabetes, higher CRP Z-score concentration was associated with higher odds of PAD and nephropathy (table 2). The differences persisted in the fully adjusted model: PAD (AOR 1.10; 95% CI 1.01 to 1.21, p=0.029) and nephropathy (AOR 1.12; 95% CI 1.04 to 1.22, p=0.004). Among participants with diabetes, higher CRP Z-score concentration was also associated with PAD and nephropathy. The associations remained statistically significant in the fully adjusted models for PAD (AOR 1.19; 95% CI 1.03 to 1.41, p=0.046), but not for nephropathy (AOR 1.13; 95% CI 0.97 to 1.31, p=0.120).

**Table 2:** Association between CRP Z-scores and microvascular/macrovascular dysfunction

	Whole cohort (n=5248)			Non-diabetes group (n=4665)			Diabetes group (n=583)		
	OR (95% CI), p value			OR (95% CI), p value			OR (95% CI), p value		
	Model 1	Model 2	Model 3	Model 1	Model 2	Model 3	Model 1	Model 2	Model 3
PAD	1.16 (1.09 to 1.25), <0.001	1.15 (1.08 to 1.24), <0.000	1.12 (1.04 to 1.21), 0.004	1.14 (1.06 to 1.23), <0.001	1.14 (1.06 to 1.23), 0.001	1.10 (1.01 to 1.21), 0.029	1.23 (1.05 to 1.43), 0.011	1.22 (1.04 to 1.43), 0.013	1.19 (1.03 to 1.41), 0.046
Nephropathy	1.15 (1.08 to 1.22), <0.001	1.12 (1.05 to 1.19), 0.001	1.13 (1.05 to 1.21), 0.001	1.13 (1.05 to 1.21), 0.001	1.10 (1.02 to 1.18), 0.014	1.12 (1.04 to 1.22), 0.004	1.17 (1.01 to 1.35), 0.040	1.17 (1.01 to 1.36), 0.037	1.13 (0.97 to 1.31), 0.120
Albuminuria	1.16 (1.08 to 1.23), <0.001	1.14 (1.07 to 1.22), <0.001	1.16 (1.08 to 1.24), <0.001	1.14 (1.06 to 1.22), 0.001	1.12 (1.04 to 1.20), 0.003	1.15 (1.05 to 1.24), 0.001	1.19 (1.02 to 1.38), 0.023	1.20 (1.03 to 1.39), 0.021	1.16 (0.99 to 1.36), 0.066
Low eGFR	1.10 (1.00 to 1.21), 0.049	1.03 (0.93 to 1.15), 0.548	1.03 (0.91 to 1.17), 0.627	1.12 (1.02 to 1.23), 0.024	1.05 (0.94 to 1.17), 0.398	1.07 (0.94 to 1.21), 0.316	0.90 (0.58 to 1.39), 0.621	0.94 (0.63 to 1.43), 0.788	0.84 (0.47 to 1.49), 0.551

Whole group analyses: model 1 was unadjusted; model 2 adjusted for age and sex; model 3 adjusted additionally for site of residence, smoking, BMI, systolic blood pressure, diabetes and LDL-cholesterol.

Subgroup (diabetes and non-diabetes) analyses: model 1 was unadjusted; model 2 adjusted for age and sex; model 3 adjusted additionally for site of residence, smoking, BMI, systolic blood pressure, and LDL-cholesterol.

BMI, body mass index; CRP, C reactive protein; eGFR, estimated glomerular filtration rate; LDL, low-density lipoprotein; PAD, peripheral artery disease.

In the analysis of the association between higher CRP Z-score concentration and the individual components of nephropathy, higher CRP Z-score concentration was associated with higher odds of albuminuria and low eGFR in the non-diabetes group. The differences persisted in the fully adjusted model for albuminuria (AOR 1.15; 95% CI 1.05 to 1.24, p=0.001), but not for low eGFR (AOR 1.07; 95% CI 0.94 to 1.21, p=0.316). Among participants with diabetes, higher CRP Z-score concentration was associated with higher odds of albuminuria, but not with low eGFR in the unadjusted model. In the fully adjusted model the association between higher CRP Z-score concentration and albuminuria was no longer statistically significant (AOR 1.16; 95% CI 0.99 to 1.36, p=0.066).

The sensitivity analysis showing the associations between CRP Z-score concentration and PAD and nephropathy in participants with CRP concentrations ≤10 mg/L is shown in table 3. In the non-diabetes group with CRP ≤10 mg/L, higher CRP Z-score concentrations were significantly associated with higher odds of PAD and nephropathy. The associations were no longer statistically significant in the fully adjusted model: PAD (AOR 1.06; 95% CI 0.93 to 1.21, p=0.372) and nephropathy (AOR 1.32; 95% CI 0.87 to 1.99, p=0.194). In participants with diabetes with CRP concentrations ≤10 mg/L, higher CRP Z-score concentration was not significantly associated with PAD or nephropathy: PAD (AOR 1.05; 95% CI 0.75 to 1.47, p=0.785) and nephropathy (AOR 1.69; 95% CI 0.77 to 3.71, p=0.189).

**Table 3:** Association between CRP Z-scores and microvascular/macrovascular dysfunction in participants with CRP concentrations ≤10 mg/L

	Whole cohort (n=4962)			Non-diabetes group (n=4430)			Diabetes group (n=532)		
	OR (95% CI), p value			OR (95% CI), p value			OR (95% CI), p value		
	Model 1	Model 2	Model 3	Model 1	Model 2	Model 3	Model 1	Model 2	Model 3
PAD	1.18 (1.07 to 1.32), 0.002	1.14 (1.03 to 1.28), 0.014	1.06 (0.94 to 1.19), 0.349	1.18 (1.05 to 1.33), 0.004	1.15 (1.02 to 1.29), 0.024	1.06 (0.93 to 1.21), 0.372	1.21 (0.92 to 1.60), 0.177	1.16 (0.87 to 1.55), 0.321	1.05 (0.75 to 1.47), 0.785
Nephropathy	1.95 (1.43 to 2.67), <0.001	1.67 (1.20 to 2.31), 0.002	1.43 (1.00 to 2.06), 0.051	1.72 (1.20 to 2.47), 0.003	1.44 (0.98 to 2.10), 0.061	1.32 (0.87 to 1.99), 0.194	1.88 (0.96 to 3.69), 0.065	2.07 (1.02 to 4.18), 0.044	1.69 (0.77 to 3.71), 0.189
Albuminuria	2.04 (1.45 to 2.88), <0.001	1.85 (1.31 to 2.63), 0.001	1.61 (1.09 to 2.38), 0.017	1.95 (1.32 to 2.89), 0.001	1.74 (1.16 to 2.59), 0.007	1.61 (1.03 to 2.51), 0.035	1.53 (0.73 to 3.24), 0.262	1.64 (0.75 to 3.59), 0.212	1.42 (0.59 to 3.40), 0.435
Low eGFR	1.30 (0.71 to 2.37), 0.397	0.95 (0.49 to 1.85), 0.890	0.74 (0.36 to 1.50), 0.400	1.00 (0.47 to 2.10), 0.993	0.68 (0.30 to 1.55), 0.363	0.61 (0.25 to 1.45), 0.260	1.56 (0.51 to 4.78), 0.433	1.96 (0.57 to 6.69), 0.283	1.20 (0.30 to 4.75), 0.796

Whole group analyses: model 1 was unadjusted; model 2 adjusted for age and sex; model 3 adjusted additionally for site of residence, smoking, BMI, systolic blood pressure, diabetes and LDL-cholesterol.

Subgroup (diabetes and non-diabetes) analyses: model 1 was unadjusted; model 2 adjusted for age and sex; model 3 adjusted additionally for site of residence, smoking, BMI, systolic blood pressure, and LDL-cholesterol.

BMI, body mass index; CRP, C reactive protein; eGFR, estimated glomerular filtration rate; LDL, low-density lipoprotein; PAD, peripheral artery disease.

## Discussion

### Key findings

Using a sample of Ghanaians, we showed that among sub-Saharan Africans higher CRP concentration is associated with higher odds of diabetes, even after adjustments for the conventional cardiovascular risk factors. Additionally, higher CRP concentration is significantly associated with higher odds of nephropathy and PAD in non-diabetes and higher odds of PAD in diabetes. The conventional cardiometabolic risk factors did not explain the associations between CRP and PAD and/or nephropathy in diabetes and non-diabetes.

### Discussion of key findings

This is the first study assessing the relationship between CRP and diabetes among a sub-Saharan African population. The results of our study in Ghanaians suggest an association between higher CRP and higher odds of diabetes in sub-Saharan Africans. A systematic review and meta-analysis by Wang *et al*^10^ had previously shown that elevated levels of inflammatory markers were significantly associated with increased risk of diabetes. The data on which this conclusion was based excluded sub-Saharan Africa origin populations. Considering the ethnic differences in the association between inflammation and diabetes, data on this association in sub-Saharan Africans are important in the quest to integrate CRP to global risk scores for diabetes.^29^ Our finding of a positive association between CRP and diabetes has confirmed earlier findings and expanded the evidence to sub-Saharan African populations. Considering the persistence of a statistically significant association between higher CRP concentration and higher odds of diabetes after excluding individuals with CRP concentrations >10 mg/L, our results could reflect the role of subclinical inflammation, instead of acute infection. Our cross-sectional analyses also suggest that a reciprocal association between inflammation and diabetes could exist as diabetes may lead to impaired immunity and increase the risk of chronic infections and/or infestations with subsequent rise in the levels of inflammatory markers.^30^

Although inflammation measured by CRP is a known risk factor for CVD, its associations with microvascular and macrovascular dysfunction in diabetes and in non-diabetes are unclear. In diabetes, most studies investigating this relationship have reported significant positive association between inflammation and vascular complications.^12 31 32^ Our results show that in sub-Saharan Africans with diabetes, higher CRP concentration is positively associated with PAD. The associations persisted after adjustments for the conventional cardiovascular risk factors. These findings resemble results previously reported in other populations.^31 32^ In this current study, we did not find a statistically significant association between CRP and nephropathy in participants with diabetes. Existing data in other ethnic groups suggest that higher CRP concentration is associated with nephropathy in diabetes.^33^ The direction of the association between CRP and nephropathy in previous studies is the same in the current study; therefore, it is possible that limited power restricted us from finding a significant association between CRP and nephropathy in diabetes.

There are limited data on the association between CRP and microvascular/macrovascular dysfunction in individuals without diabetes. Our study shows that in sub-Saharan Africans without diabetes, higher CRP concentration is significantly associated with PAD and nephropathy. This finding highlights the role of inflammation in the pathogenesis of microvascular/macrovascular dysfunction, independent of hyperglycemia.^17 34^ This has important implications in low-to-middle-income regions like sub-Saharan Africa, where in addition to the growing burden of obesity and diabetes there is a high burden of proinflammatory conditions, including chronic or recurrent infections and infestations.^6 11 35 36^ Chronic intravascular infections and infestations may trigger the inflammatory pathways, predisposing the macrovasculature and microvasculature to accelerated atherosclerosis.^17 37^ Also, chronic extravascular infections can enhance extravascular production of inflammatory cytokines that may accelerate the process of atherosclerosis.^17^ The conventional cardiometabolic risk factors did not significantly attenuate the association between CRP and PAD or nephropathy in non-diabetes. This further highlights the fact that the inflammatory pathways driving microvascular and macrovascular dysfunction may not be fully dependent on the conventional cardiovascular risk factors. Indeed, we have previously reported that the higher rates of microvascular and macrovascular dysfunction in sub-Saharan Africans living in Africa compared with their peers in Europe are not fully explained by glycemic control or the conventional cardiovascular risk factors.^38 39^

Overall, our results support the idea that some process related to persistent inflammation is associated with microvascular and macrovascular dysfunction, although the clinical utility of this remains unclear, especially in the setting of low-income to middle-income countries where the likelihood of low-grade inflammation from non-hyperglycemic causes including chronic infections and infestations is high.^36^ Based on the CANTOS trial (Canakinumab Anti-Inflammatory Thrombosis Outcomes Study), anti-inflammatory therapy targeting the interleukin-1β pathway significantly reduced macrovascular events.^40^ Although not previously investigated, the finding from the CANTOS trial may suggest that interventions aimed at controlling low-grade inflammation may be valuable in curbing the rates of microvascular and macrovascular dysfunction. This may include relatively less expensive interventions like control of chronic infections and infestations. Another clinical significance of our results is the potential of using CRP as a marker for assessing or monitoring the risk of developing vascular dysfunction in individuals with and without diabetes. This could aid early detection of vascular dysfunction, improve quality of life and reduce healthcare-related costs through preventive interventions.

### Limitations

Our study has some limitations. First, we used hs-CRP as the marker of inflammation. hs-CRP does not necessarily reflect the degree of inflammation of individual tissues. Additionally, CRP is only one marker of inflammation and other markers such as fibrinogen and D-dimer were not measured in our study. Second, we did not have data on existing acute infections or infestations; however, we excluded participants with CRP concentrations >10 mg/L in a sensitivity analysis. Third, the sample size among individuals with diabetes was relatively small, limiting the power to detect associations between CRP and vascular complications in diabetes. Fourth, the microvascular dysfunction of retinopathy and neuropathy, as well as the macrovascular dysfunction of coronary artery disease and cerebrovascular disease, were not assessed. Fifth, we did not perform the 2-hour postload plasma glucose in identifying participants with diabetes; this could have underdiagnosed participants with diabetes.^24^ Finally, the duration of diabetes was not included as a covariate in the multivariable analysis for participants with diabetes because many study participants did not provide this information.

## Conclusion

In our study, higher CRP concentration was associated with higher odds of diabetes. Higher CRP concentration was also associated with higher odds of PAD and nephropathy in non-diabetes and higher odds of PAD in diabetes. Our data from this first study on CRP in relation to diabetes and microvascular and macrovascular dysfunction in a sub-Saharan African population suggest that CRP could be explored as a potential marker to identify sub-Saharan African patients with diabetes at risk for macrovascular complications. Additionally, our finding of an association with higher odds of diabetes and both microvascular and macrovascular dysfunction in non-diabetes individuals implies that CRP could play a role in the assessment of risk before diabetes diagnosis, for example, in pre-diabetes individuals. In both individuals with and without diabetes, interventions aimed at controlling inflammation may help reduce the risk of diabetes and microvascular and macrovascular dysfunction.
