# Supplementary material for: Association between C reactive protein and microvascular and macrovascular dysfunction in sub-Saharan Africans with and without diabetes: the RODAM study
Source: BMJ Open Diabetes Res Care. 2020 Jul 14;8(1):e001235. doi: 10.1136/bmjdrc-2020-001235 (PMC7365428; doi:10.1136/bmjdrc-2020-001235)
Supplement: Supplementary data [file bmjdrc-2020-001235supp001.pdf]

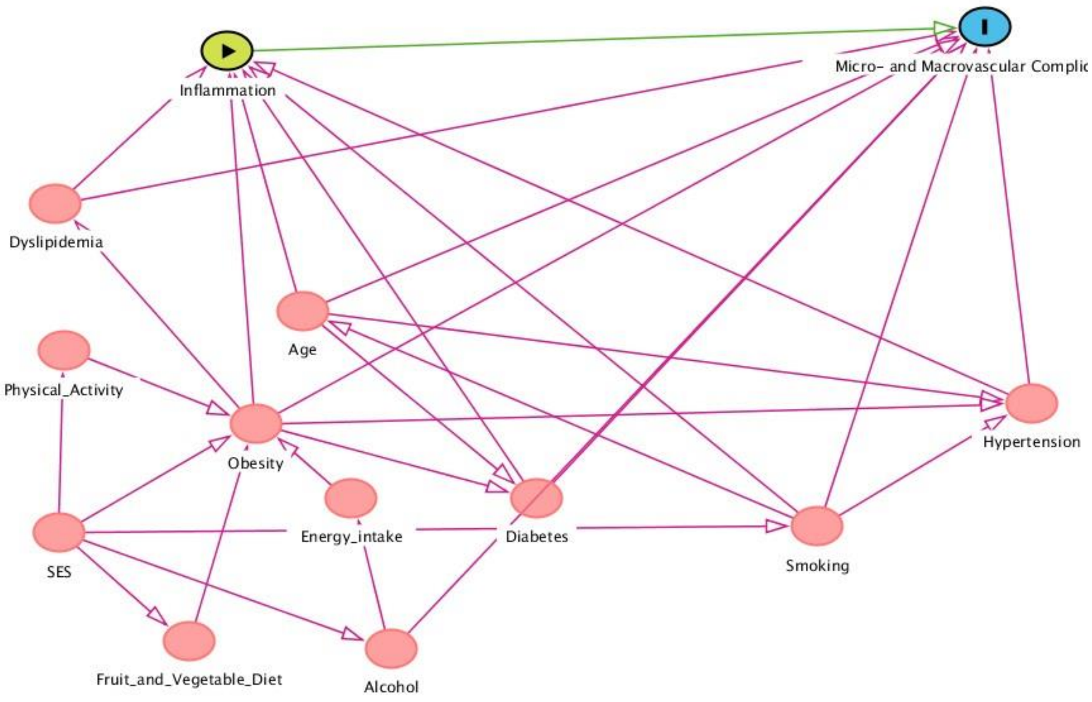

Supplementary Figure 1: Directed acyclic graph for determining the minimal sufficient adjustment sets for estimating the total effect of CRP on Micro-and macrovascular disease.

Legend: 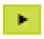 = exposure; 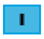 = outcome; 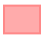 ancestor of exposure *and* outcome; = causal path.

Definition of abbreviations: SES = socioeconomic status.

Link to DAG: dagitty.net/mGETVa3
